# Respiratory performance in Duchenne muscular dystrophy: Clinical manifestations and lessons from animal models

**DOI:** 10.1113/EP091967

**Published:** 2024-07-18

**Authors:** Rebecca Delaney, Ken D. O'Halloran

**Affiliations:** ^1^ Department of Physiology University College Cork Cork Ireland

**Keywords:** control of breathing, D2.*mdx* mice, Duchenne muscular dystrophy, *mdx* mice, peak inspiratory pressure

## Abstract

Duchenne muscular dystrophy (DMD) is a fatal genetic neuromuscular disease. Lack of dystrophin in skeletal muscles leads to intrinsic weakness, injury, subsequent degeneration and fibrosis, decreasing contractile function. Dystropathology eventually presents in all inspiratory and expiratory muscles of breathing, severely curtailing their critical function. In people with DMD, premature death is caused by respiratory or cardiac failure. There is an urgent need to develop therapies that improve quality of life and extend life expectancy in DMD. Surprisingly, there is a dearth of information on respiratory control in animal models of DMD, and respiratory outcome measures are often limited or absent in clinical trials. Characterization of respiratory performance in murine and canine models has revealed extensive remodelling of the diaphragm, the major muscle of inspiration. However, significant compensation by extradiaphragmatic muscles of breathing is evident in early disease, contributing to preservation of peak respiratory system performance. Loss of compensation afforded by accessory muscles in advanced disease is ultimately associated with compromised respiratory performance. A new and potentially more translatable murine model of DMD, the D2*.mdx* mouse, has recently been developed. Respiratory performance in D2*.mdx* mice is yet to be characterized fully. However, based on histopathological features, D2*.mdx* mice might serve as useful preclinical models, facilitating the testing of new therapeutics that rescue respiratory function. This review summarizes the pathophysiological mechanisms associated with DMD both in humans and in animal models, with a focus on breathing. We consider the translational value of each model to human DMD and highlight the urgent need for comprehensive characterization of breathing in representative preclinical models to better inform human trials.

## Abstract (highlights)

- **What is the topic of this review?**
  We review current understanding of the respiratory phenotype in Duchenne muscular dystrophy (DMD), a fatal genetic neuromuscular disease.
- **What advances does it highlight?**
  Diaphragm muscle dystropathology and contractile dysfunction present early in dystrophic disease, yet respiratory system performance is maintained by compensatory mechanisms until late‐stage advanced disease. Loss of compensation afforded by accessory muscles of breathing contributes to respiratory morbidity in canine and murine models of DMD, mirroring the human disease. The D2.*mdx* mouse appears convenient for the study of the complex respiratory phenotype associated with dystrophin deficiency, and assessments of new therapeutics in this model might be translatable to human DMD.

## 1 DUCHENNE MUSCULAR DYSTROPHY

### 1.1 Introduction

**FIGURE 1:**
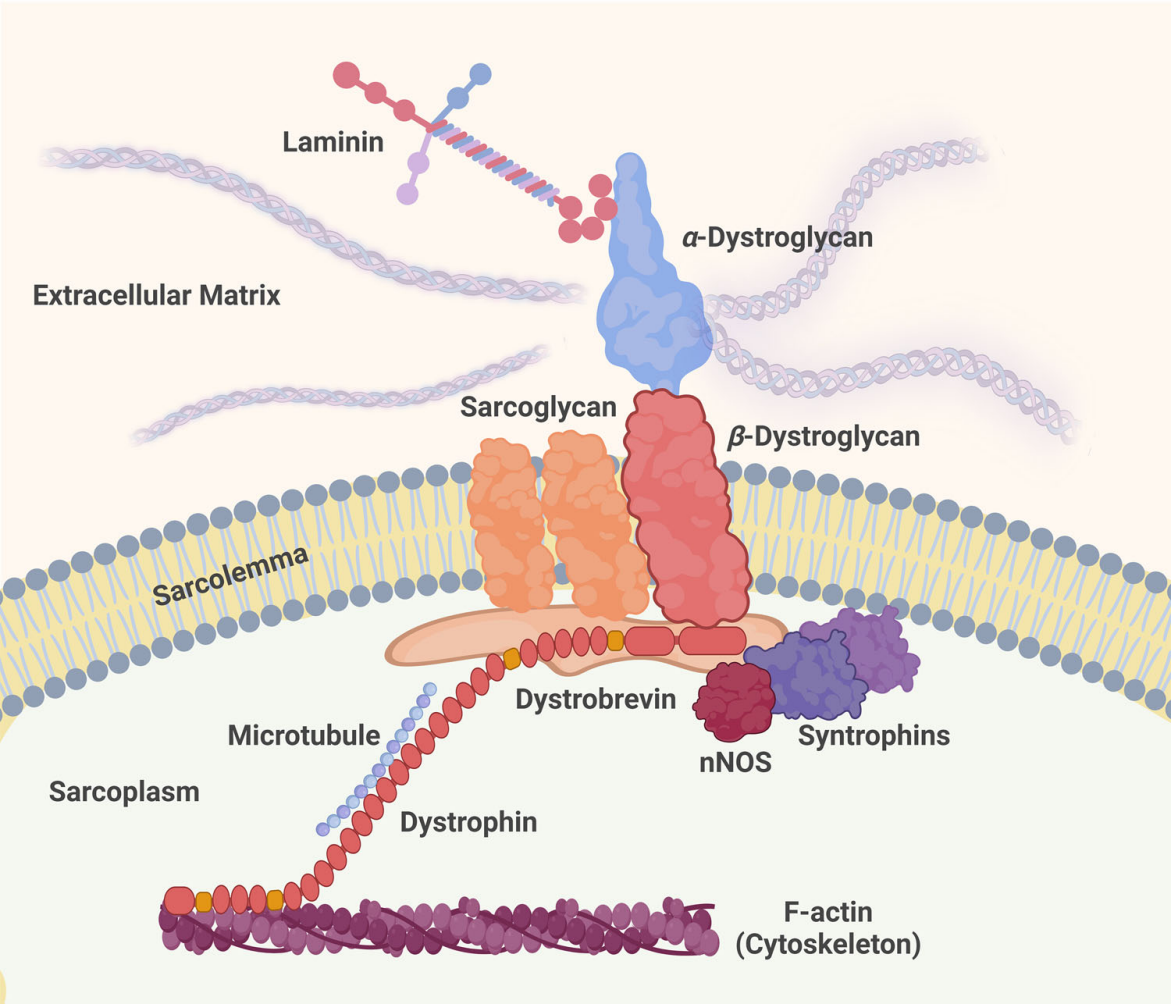
Schematic diagram of the dystrophin–glycoprotein complex situated at the sarcolemma. Dystrophin binds to the actin cytoskeleton via the N‐terminus. The sarcospan–sarcoglycan sub‐complex forms part of the dystrophin–glycoprotein complex. Syntrophins are adaptor proteins that link dystrophin with signalling molecules. α‐Dystroglycan is a laminin‐binding protein that binds to the transmembrane protein, β‐dystroglycan. Dystrophin is a microtubule‐associated protein. Created with BioRender.com.

Duchenne muscular dystrophy (DMD) is a fatal X‐linked neuromuscular disease. It is characterized by a complete absence of the 427 kDa protein, dystrophin. Dystrophin is localized at the sarcolemma of skeletal and cardiac muscle (Figure 1). It plays a role in maintaining muscle integrity during repeated oscillations of contraction and relaxation (Wong et al., 2020). Dystrophin links the internal cytoskeleton to the extracellular matrix, as part of the dystrophin glycoprotein complex (DGC). Muscles that completely lack dystrophin become susceptible to contraction‐mediated injury and, ultimately, muscle fibre loss. Fibre necrosis is associated with an influx of inflammatory cells, such as macrophages and CD4^+^ lymphocytes (Blake et al., 2002). Excessive fibrosis and fat deposition occur in dystrophic muscles because of repeated injury, which alters their architecture, forming scar tissue, which further impairs the contractility of the muscles (Kharraz et al., 2014).

Duchenne muscular dystrophy occurs mainly in boys, with a prevalence of 1 in 3500–5000 live births. It is the most frequently occurring muscular dystrophy in paediatric populations (Wang et al., 2018). Developmental milestones are significantly delayed in DMD. The disease initially manifests as muscle weakness in the lower extremities at ∼2–3 years of age. Patients have problems walking, running and jumping and may also struggle to carry out fine motor movements. Gowers’ sign is a classic feature of DMD, where patients must use their upper limbs to pull themselves up from a squatting position. This indicates loss of strength of the hip and thigh muscles (Wallace & Newton, 1989). Muscle weakness then starts to present in the upper extremities. The rapidly progressive nature of DMD unfortunately leads to complete loss of ambulation by ∼12 years of age. In the late teenage years, muscle weakness starts to become evident in the muscles involved in respiration, and impairments start to arise, such as hypoventilation and aspiration (MacKintosh et al., 2020). Death most commonly occurs by respiratory failure in their twenties, owing to wasting of the muscles critical in the control of breathing (Aliverti et al., 2015). However, interventions such as mechanical ventilation can help to extend the life expectancy of people with DMD into the fourth decade (Ishikawa et al., 2011). Advances in respiratory care and management in DMD extend the lifespan such that cardiomyopathy progresses and therefore becomes the predominant cause of death (Lechner et al., 2023). Unfortunately, there is no curative therapy yet available for people with DMD.

### 1.2 Aetiology and pathophysiology of Duchenne muscular dystrophy

Duchenne muscular dystrophy is caused by a mutation in the largest gene in the human genome, *DMD*, which contains 79 exons (Koenig et al., 1987). Large deletions in this gene account for most of the mutations that occur in DMD cases (Muntoni et al., 2003). Point, nonsense mutations and large duplications also occur in *DMD*, but present at a lower frequency than large deletions (Bladen et al., 2015). Animal models of DMD, particularly the dystrophin‐deficient *mdx* mouse model, have provided great insights into the pathophysiology of DMD. In the early stages of disease, muscle fibre necrosis occurs in groups, surrounded by an abundance of inflammatory mediators. The characteristic fibre necrosis is mediated by an influx of calcium into the hyperpermeable sarcolemma and leads to activation of proteases (Zhou & Lu, 2010). Cycles of degeneration and regeneration occur; however, the regenerated muscle fibres still lack dystrophin. Therefore, the muscle cannot adequately repair itself and restore its function, and there is a decrease in the amount of contractile muscle fibres. A hallmark of attempted muscle regeneration seen in muscle biopsies are centrally nucleated fibres (Deconinck & Dan, 2007). As the disease progresses, satellite cells, the muscle stem cells, become overwhelmed and eventually deteriorate (Dumont & Rudnicki, 2016). Muscle fibres are replaced by fibrotic and adipose tissue, which reduce contractility, and therefore, impair the function of the muscles.

The myopathy associated with DMD has severe complications for limb and respiratory muscles, particularly the diaphragm.

### 1.3 Diaphragm dysfunction in Duchenne muscular dystrophy

The diaphragm is constantly active throughout life, from our very first breath at birth to our very last at the end of life. Therefore, dystrophin in the diaphragm muscle is essential to protect it from repeated mechanical stress associated with a high duty cycle. The characteristic fibrosis that develops in DMD severely impacts the normal functioning of the diaphragm. Along with the accumulation of fat and many inflammatory cells, the diaphragm becomes pseudo‐hypertrophic and loses its elastic properties (Laviola et al., 2018). MRI studies have identified evidence of diaphragm compromise in DMD patients, characterized by reduced capacity to move during respiration (Mankodi et al., 2017; Pennati et al., 2020), negatively affecting pulmonary ventilation. As the disease progresses, the diaphragm muscle, along with other critical muscles of respiration, weaken further, compromising respiratory performance, which eventually leads to complete respiratory failure, owing to increasing respiratory load and the inability to overcome this load (Lo Mauro & Aliverti, 2016). The tension–time index of the diaphragm, a measure of diaphragm endurance, tends to decrease in the late teenage years in DMD (Khirani et al., 2014).

## 2 THE RESPIRATORY PHENOTYPE IN DUCHENNE MUSCULAR DYSTROPHY

### 2.1 Ventilatory insufficiency in Duchenne muscular dystrophy

**FIGURE 2:**
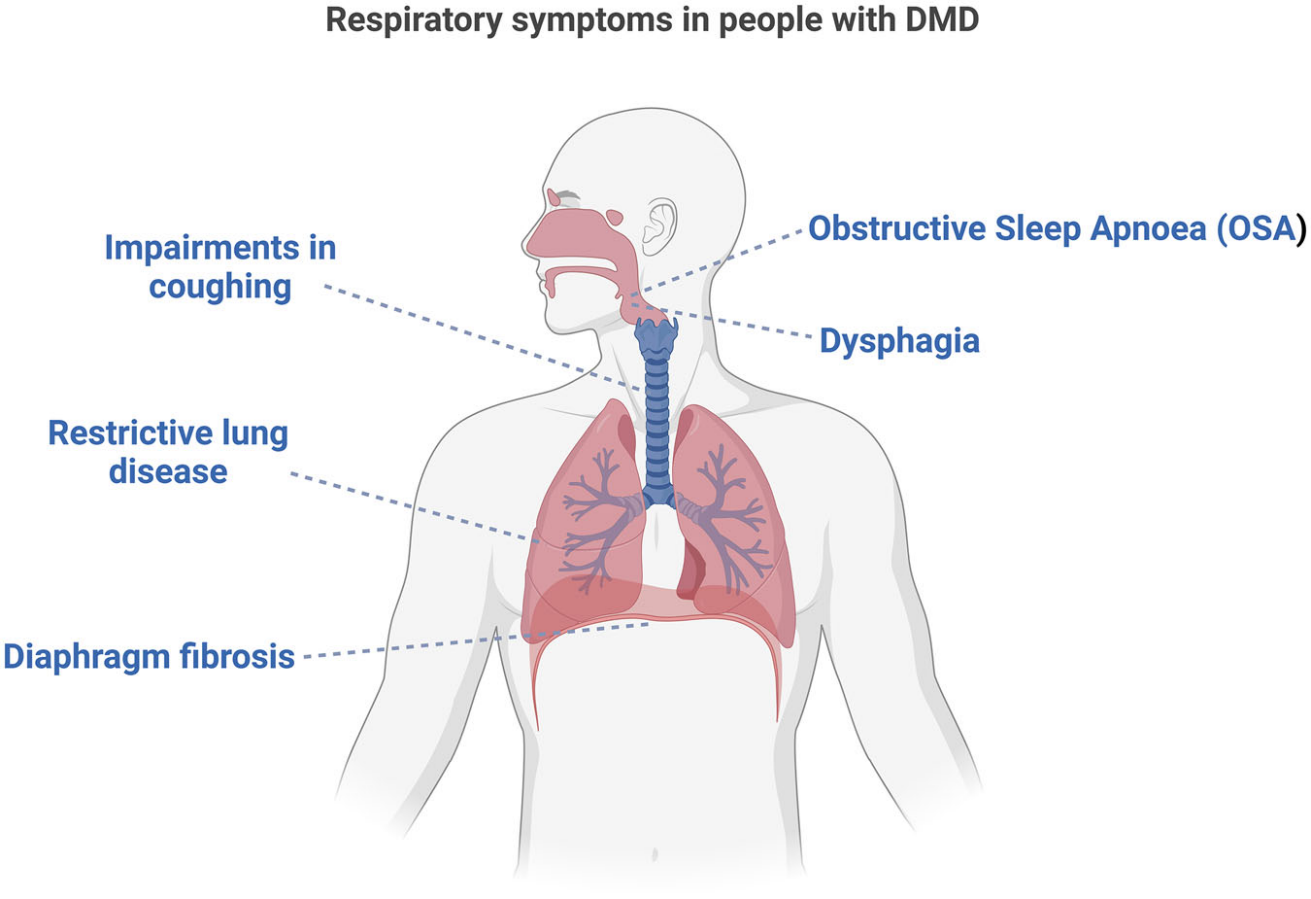
Main features of Duchenne muscular dystrophy that contribute to respiratory system morbidity. Created with BioRender.com.

Atrophy and weakness of the respiratory muscles has serious implications for the DMD patient (Figure 2). Hypoventilation, dyspnoea, sleep‐disordered breathing, atelectasis, aspiration pneumonia and impairments in coughing are all common features of the disease (Mhandire et al., 2022). The progressive nature of DMD eventually culminates in death by cardiorespiratory failure. The extent of respiratory muscle weakness largely determines the patient's prognosis. DMD causes restrictive lung disease, along with decreased chest wall compliance and decreased airway clearance, as measured by spirometry (Lo Mauro, 2016). The characteristic fibrotic deposition in the diaphragm, intercostal muscles and accessory muscles of respiration causes them to become stiff, and they lose their ability to contract fully. The symptoms associated with impairments in breathing may not become evident until there are changes in breathing at rest, which occurs in the later stages of the disease. Therefore, it is vital that pathophysiological changes in ventilatory capacity are identified as early as possible in the patient, in order that interventions can be introduced to improve respiratory capacity and, hopefully, improve their quality of life.

### 2.2 Forced vital capacity, maximal inspiratory and expiratory pressures in Duchenne muscular dystrophy

The routine testing of pulmonary function by measuring physiological parameters is essential in the management of DMD. Forced vital capacity (FVC) is the maximum amount of air that can be expired fully after maximal inhalation. Therefore, it measures the function of both inspiratory and expiratory muscles. In the patient's early life, FVC will increase gradually each year as the lung grows until ∼10 years of age, when peak FVC is reached (Table 1; Khirani et al., 2014; Mayer et al., 2015). In the late teenage years in DMD, FVC begins to rapidly decline at a rate of 5%–8% per year. The age of death in DMD patients attributable to ventilatory failure is variable. However, it has been reported that once FVC falls below 1 L, it is a strong indicator of morbidity for the patient (Phillips et al., 2001). The decline in FVC values is correlated with a reduction in maximal inspiratory pressure, which reflects the efficacy of inspiratory muscle performance. The maximal inspiratory pressure also reaches a peak in the early years of life and declines steadily at the end of the first decade (Table 2; Figure 1; Gayraud et al., 2010; Matecki et al., 2001). A similar pattern for maximal expiratory pressures is evident in DMD. It was found that maximal expiratory pressure reaches a peak value in the mid‐teens, and then rapidly decreases in the late teenage years and early twenties (Hahn et al., 1997). This reflects the progressive expiratory muscle weakness that occurs in DMD, and largely affects the ability of the patient to perform manoeuvres, such as coughing (essential for airway clearance), that depend on expiratory muscle strength.

**TABLE 1:** Forced vital capacity measurements in Duchenne muscular dystrophy patients.

Forced vital capacity	Phillips et al. (2001)	Mayer et al. (2015)	Khirani et al. (2014)
Age at peak value of forced vital capacity, years	Between 13 and 15	10	Between 13 and 14
Mean decline of forced vital capacity per year after peak, %	−8.0	−5.0 ± 0.7	−4.9 ± 4.4

**TABLE 2:** Maximal inspiratory pressure measurements in Duchenne muscular dystrophy patients and control participants.

Maximal inspiratory pressure, cmH_2_O	Hahn et al. (1997)	Matecki et al. (2001)
Mean in DMD patients	49.1 ± 13.4	52.5 ± 11
Mean in healthy control participants	Not reported	112 ± 20

### 2.3 Sniff nasal inspiratory pressure in Duchenne muscular dystrophy

Sniff nasal inspiratory pressure (SNIP) is a physiological measure of inspiratory muscle strength. It is non‐invasive and easily performed in young patients with DMD, given that spirometry can often be difficult to perform because it requires complete cooperation from the patient. Sniff nasal inspiratory pressure has therefore been deemed to be a more reliable measure of respiratory function, because it can detect changes in muscle weakness before the onset of changes in FVC and other respiratory parameters. It is vital that the earliest disturbances in respiratory performance are detected, because they determine prognosis and can guide earlier treatment or intervention, culminating in better outcomes for the patient. In a study by Nève et al. (2013), 23 patients with DMD had declining inspiratory muscle strength detectable at a mean age of 10 years old, whereas vital capacity began to decline at ∼12 years of age. It was also demonstrated that those who required ventilation at an earlier age, on average, had lower SNIP values in comparison to patients who did not start receiving ventilation until a later age. Therefore, performing SNIP measurements as early as possible in patients can help to identify prognostic markers. Combining maximal inspiratory pressure and maximal expiratory pressure with SNIP is crucial in determining overall respiratory performance in the patient.

### 2.4 Non‐ventilatory behaviours in Duchenne muscular dystrophy

Respiratory muscle weakness, especially in expiratory muscles of the abdominal wall, leads to impairments in coughing manoeuvres and clearance of airway secretions, which has detrimental effects for DMD patients. The inability to cough can lead to recurrent infections and atelectasis and it increases the risk of pneumonia, which is fatal when it occurs in conjunction with respiratory muscle failure (LoMauro et al., 2014). Scoliosis, which is evident in a large number of patients, can also affect the ability of the patient to cough and it reduces the reserve volume of the lung (Birnkrant, 2002). The characteristic chest wall stiffness and decreased lung compliance compromise the inspiratory phase of coughing, and declining FVC values are associated with lower peak cough flow values (LoMauro et al., 2014). The normal value for peak cough flow is 300 L/min. Gauld and Boynton (2005) observed that when peak cough flow falls below 270 L/min in DMD patients, they have reduced capacity to clear airway secretions during respiratory infections. Cough‐assisted technologies have been used to aid in airway clearance for patients whose peak cough flow is <160 L/min (Camela et al., 2019). The oropharyngeal muscles also weaken in DMD, and a subset of patients experience dysphagia, an impairment in swallowing. Dysphagia can lead to aspiration pneumonia, which is a common cause of death in DMD (Toussaint et al., 2016).

### 2.5 Sleep‐disordered breathing in Duchenne muscular dystrophy

Obstructive sleep apnoea (OSA) is the most common type of sleep‐disordered breathing that occurs in patients with DMD (Bamaga & Alqarni, 2023). During sleep, there are challenges to respiratory control, owing to blunted reflexes, and increased risk of airway collapse, owing to state‐dependent reductions in upper airway muscle tone. Obstructive sleep apnoea is characterized by periods of breathing cessation (apnoea) or reduced breathing (hypopnoea) and subsequent oxygen desaturation. Obstructive sleep apnoea in patients with DMD can occur as early as 12 years of age (Suresh et al., 2005), with airway obstructions commonly manifesting during rapid eye movement (REM) sleep (Sawnani et al., 2015). Upper airway resistance increases during REM sleep owing to hypotonia of the pharyngeal muscles. DMD patients exhibit progressive weakening of skeletal muscles of the face, neck and upper airway. This increases the propensity for upper airway collapse because airway patency cannot be maintained adequately. Obesity is a risk factor for the development of OSA (Romero‐Corral et al., 2010). Long‐term glucocorticoid therapy in patients with DMD often leads to weight gain, and excess fat deposition can contribute to narrowing of the pharyngeal airway (Sawnani et al., 2015). The severity of OSA is measured by the number of periods of apnoea and hypopnoea that occur each hour, scored as the apnoea–hypoponea index (Asghari & Mohammadi, 2013). Patients with DMD have been shown to present with moderate OSA, with an average of 18 apnoea/hypopnoea events per hour (Bersanini et al., 2012).

Reductions in blood oxygenation occur as a direct consequence of the obstruction to airflow, and this leads to a rapid arousal. Alternating periods of apnoea and arousal are evident in DMD patients even in the absence of symptoms associated with poor sleep (Smith et al., 1988). The patient may not be aware that they are experiencing sleep‐disordered breathing, which can be detrimental to their health, because sleep‐disordered breathing is associated with hypertension, increased risk of stroke and excessive daytime sleepiness (Somers et al., 2008). Hypoventilation, hypercapnia and hypoxaemia present first during sleep in DMD, while blood gas levels remain normal during the day (Smith et al., 1988). As the disease progresses, diurnal hypercapnia occurs, and death typically ensues 1 year following this presentation (Simonds et al., 1998). Observation of breathing during sleep throughout the disease is an important component of patient care in DMD. Obstructive sleep apnoea in DMD can be treated adequately by nocturnal non‐invasive ventilation to prevent fluctuations in blood O_2_ and CO_2_ levels (LoMauro et al., 2017).

### 2.6 Current treatments for Duchenne muscular dystrophy

The use of mechanical ventilation in the management of DMD has been crucial in extending the lifespan of patients from their early twenties to forties (Eagle et al., 2002). Non‐invasive ventilation reduces the mechanical load on degenerating respiratory muscles and slows the progression of the disease (Toussaint et al., 2006). Glucocorticoids, such as prednisone, have been used in patients with DMD, because they dampen the large inflammatory response that occurs in muscle (Zhang & Kong, 2021). Glucocorticoids reduce the annual decline of FVC, which largely predicts mortality in patients (McDonald et al., 2018). However, long‐term administration of glucocorticoids is not optimal because it can lead to Cushing's disease, which has deleterious effects on growth and bone health in patients. Moreover, chronic glucocorticoid use leads to muscle wasting. Antisense oligonucleotides have been used in recent years to restore dystrophin. Eteplirsen, for exon 51 skipping, has been shown to attenuate deteriorating respiratory performance owing to a reduction in the rate of annual FVC decline (Kinane et al., 2018). Gene therapy is currently being investigated in numerous clinical trials, using adeno‐associated virus (AAV) technology to deliver a smaller form of dystrophin, called ‘micro‐dystrophin’ (Chamberlain & Chamberlain, 2017). However, there is a paucity of clinical trials that assess the efficacy of gene therapies on respiratory function (Mhandire et al., 2022). Indeed, this has not been addressed adequately at the preclinical level.

## 3 ANIMAL MODELS OF DUCHENNE MUSCULAR DYSTROPHY

### 3.1 Murine and canine models

The use of animals in preclinical research of DMD has been vital in furthering our understanding of the disease. With murine models, such as the *mdx* (dystrophin‐deficient) model, histopathological factors of DMD have been well characterized, along with the time at which certain features arise. The low cost and the relative ease in manipulating genetic backgrounds in murine models have led to their extensive use in DMD research. Measurements of physiological parameters have been conducted in the *mdx* and *mdx/utrn* ^−/−^ (dystrophin/utrophin‐deficient) mouse models, demonstrating the progressive functional impairments that occur in DMD. Canine models, such as the golden retriever muscular dystrophy (GRMD) model, have been used particularly in testing of treatments for DMD. Animal models of DMD should ideally recapitulate the dystrophic human phenotype to ensure successful translational research. A relatively new murine model of DMD, the D2.*md*x mouse, is currently being characterized and might be especially useful in the testing of emerging and established treatments for human DMD.

### 3.2 The *mdx* mouse model

The *mdx* mouse model was discovered after a spontaneous mutation arose in an inbred C57BL/10 colony of mice (Bulfield et al., 1984). A missense mutation occurred in exon 23 of the DMD gene, leading to the absence of dystrophin (Sicinski et al., 1989). As early as 3 weeks of age, pathological changes begin to appear in the limb muscles of *mdx* mice. Like the human phenotype of DMD, variation in muscle fibre sizes, atrophy attributable to muscle fibre degeneration and infiltration of immune cells are evident at this stage. However, extensive necrosis of muscle fibres in the gastrocnemius and soleus muscles reaches a peak at ∼3–4 weeks and plateaus thereafter, producing a milder dystrophic phenotype in limb muscles in comparison to the progressive degeneration in humans with DMD (DiMario et al., 1991). It has been hypothesized that satellite cells in muscle, which function as stem cells, contribute to the muscle regeneration in the limb muscles of the *mdx* mouse, because they are still largely effective in aged *mdx* mouse models (Boldrin et al., 2015). In contrast to the limb muscles, pathology in the diaphragm muscle emerges early (Coirault et al., 2003; O'Halloran et al., 2023) and is progressively enhanced, recapitulating the progressive weakening of the diaphragm with age seen in humans with DMD (Stedman et al., 1991). Therefore, testing the efficacy of therapies in preclinical trials in *mdx* mice is relevant owing to the resemblance of the disease course to the human dystrophic diaphragm.

Following a disruption in the balance between muscle degeneration and regeneration in the diaphragm, a considerable amount of fibrosis follows, with deposition of collagen, rendering the muscle stiff, with loss of its elastic properties. However, it has been shown that considerable reductions in maximal isometric tension occur in the *mdx* mouse diaphragm in the first month of life, ahead of excessive collagen deposition (Coirault et al., 2003; O'Halloran et al., 2023), highlighting intrinsic impairment in muscle fibre function. Reductions in the number of cross‐bridges, which are crucial components of effective muscle contraction, are evident in *mdx* mice along with shorter cycles. It has been hypothesized that early alterations to myosin molecules contribute to the reduced contractility of the *mdx* diaphragm along with fibrosis, which occurs at a later stage. An increased complement of type IIA myosin heavy chain fibres in comparison to control mice might also contribute to the reduction in peak force production, which continues to decline with increasing age in the *mdx* mice (Burns et al., 2017; Coirault et al., 1999; Petrof et al., 1993).

Characterization of respiratory performance has been determined in *mdx* mice through in vivo measurement of respiratory parameters. Similar to the human phenotype, respiratory capacity declines with age in *mdx* mice, purportedly owing to progressive weakening of the diaphragm (Table 3; Mosqueira et al., 2013). Of note, Ishizaki et al. (2008) observed a rise in respiratory rate with age in *mdx* mice, along with a significant decrease in tidal volume compared with control mice, suggesting that the tachypnoea is a compensatory mechanism owing to increased load on the diaphragm muscle and progressive decline in muscle function. Burns et al. (2017) reported that *mdx* mice hypoventilate, because they show a reduced ventilatory equivalent for carbon dioxide as early as 8 weeks of age, with additional evidence from arterial blood gas analysis supportive of a compensated respiratory acidosis. At 8 weeks of age, *mdx* mice have a significantly lower minute ventilation than wild‐type mice, owing to reduced tidal volume (Table 3; Burns et al., 2018, 2017). There are conflicting reports on the control of breathing in *mdx* mice. Other studies have reported no significant differences in basal respiratory parameters between *mdx* and control mice (Table 3; Burns, Murphy et al., 2019; Gosselin et al., 2003; Maxwell et al., 2023). The inconsistencies in results might be attributable to differences in study protocols or heterogeneity in disease expression.

**TABLE 3:** Respiratory patterns in *mdx* mice during normoxic conditions.

Reference	Respiratory rate	Tidal volume	Minute ventilation
Gosselin et al. (2003)	No significant differences between 7‐month‐old *mdx* mice and age‐matched controls	No significant differences between 7‐month‐old *mdx* mice and age‐matched controls	No significant differences between 7‐month‐old *mdx* mice and age‐matched controls
Ishizaki et al. (2008)	Two‐ and 4‐month‐old *mdx* mice: lower than age‐matched control mice. Seven‐month‐old *mdx* mice: significantly higher than age‐matched control mice	Seven‐month‐old *mdx* mice: significantly lower than control mice	No significant differences between *mdx* mice and controls at 7 months
Huang et al. (2011)	Three‐month‐old *mdx* mice: significantly lower than age‐matched control mice. Six‐month‐old *mdx* mice: significantly lower than age‐matched control mice	Three‐month‐old *mdx* mice: significantly lower than age‐matched control mice. Six‐month‐old *mdx* mice: significantly lower than age‐matched control mice	Three‐month‐old *mdx* mice: significantly lower than age‐matched control mice. Six‐month‐old *mdx* mice: significantly lower than age‐matched control mice
Mosqueira et al. (2013)	Six‐ and 7‐month‐old *mdx* mice: significantly lower than age‐matched control mice	Six‐ and 7‐month‐old *mdx* mice: significantly lower than age‐matched control mice	Six‐ and 7‐month‐old *mdx* mice: significantly lower than age‐matched control mice
Burns et al. (2017)	No significant difference between 8‐week‐old *mdx* mice and age‐matched controls	Eight‐week‐old *mdx* mice: significantly lower than control mice	Eight‐week‐old *mdx* mice: significantly lower than control mice
Burns et al. (2018)	No significant difference between 8‐week‐old *mdx* mice and age‐ matched controls	Eight‐week‐old *mdx* mice: significantly lower than control mice	Eight‐week‐old *mdx* mice: significantly lower than control mice
Burns, Murphy et al. (2019)	No significant difference between 8‐week‐old *mdx* mice and age‐matched controls	No significant difference between 8‐week‐old *mdx* mice and controls	No significant difference between 8‐week‐old *mdx* mice and controls
Maxwell et al. (2023)	Not reported	Not reported	No significant difference between 4‐month‐old *mdx* mice and age‐matched controls

Data regarding the ability of *mdx* mice to respond adequately to hypercapnia are mixed. Despite distinct pathological changes in the diaphragm emerging early in *mdx* mice, increases in respiratory rate, tidal volume and minute ventilation in response to hypercapnic stimuli are reported as being similar to control mice, sufficiently maintaining blood‐gas homeostasis (Burns, Murphy et al., 2019; Gayraud et al., 2007). However, there are other reports in young *mdx* mice of decreased ventilatory responsiveness to hypercapnia (Burns et al., 2018) and decreased ventilation in hypercapnia but with preserved ventilatory responsiveness, determined by the change in ventilation from baseline (Burns et al., 2017) (Table 4). In late dystrophic disease, the capacity to increase ventilation in response to hypercapnic conditions is markedly decreased in *mdx* mice in comparison to control mice (Table 4). It appears that after 5 months of age in *mdx* mice, the ability to alter ventilatory patterns in response to changes in CO_2_ concentrations is significantly reduced. Interestingly, the diaphragm muscle retains considerable force reserve despite injury and fibrosis, which probably continues to support ventilatory capacity given the observation that ventilatory responses to chemoactivation can be achieved with <50% of peak force (Mantilla et al., 2010). Thus, in early dystrophic disease, the significant reserve capacity of the diaphragm is likely to be sufficient to maintain ventilation in *mdx* mice that is equivalent to wild‐type mice. Ventilatory responses to chemoreceptor stimulation in models of DMD are relevant to patients with DMD, who often have sleep disordered breathing and experience hypercapnia and hypoxaemia, which can, potentially, worsen their pathology. Establishing the age/disease stage at which certain respiratory defects occur in *mdx* mouse models is crucial for ensuring adequate translational applicability to humans with DMD.

**TABLE 4:** Respiratory patterns in *mdx* mice during various hypercapnic conditions.

Reference	Respiration rate	Tidal volume	Minute ventilation
Gosselin et al. (2003) 7% CO_2_	Significantly lower in 10‐12‐month‐old *mdx* mice compared with control mice	Significantly lower in 10‐12‐month‐old *mdx* mice compared with control mice	Significantly lower in 10‐12‐month‐old *mdx* mice compared with control mice
Gayraud et al. (2007) 8% CO_2_	No significant difference between 5‐month‐old *mdx* mice and control mice. Significantly lower in 16‐month‐old *mdx* mice compared with control mice	No significant difference between 5‐month‐old *mdx* mice and control mice. Significantly lower in 16‐month‐old *mdx* mice compared with control mice	No significant difference between 5‐month‐old *mdx* mice and control mice. Significantly lower in 16‐month‐old *mdx* mice compared with control mice
Burns et al. (2017) 5% CO_2_	Not reported	Not reported	Significantly lower in 8‐week‐old *mdx* mice compared with control mice
Burns et al. (2018) 5% CO_2_	Not reported	Not reported	Significantly lower in 8‐week‐old *mdx* mice compared with control mice
Burns, Murphy et al. (2019) Graded hypercapnia (2%, 4% and 6%) and hypercapnic hypoxia: 10% O_2_, 6% CO_2_	No significant difference between 8‐week‐old *mdx* mice and control mice	No significant difference between 8‐week‐old *mdx* mice and control mice	No significant difference between 8‐week‐old *mdx* mice and control mice

In the study by Mosqueira et al. (2013), *mdx* mice displayed a significant decrease in respiratory rate during mild hypoxic challenges and displayed reduced phrenic nerve activity. The phrenic nerves innervate the diaphragm, and phrenic neural drive increases in response to perturbations in blood‐gas homeostasis, as detected by chemoreceptors such as the carotid body and central chemoreceptors. Interestingly, Mosqueira et al. (2013) also found that dystrophin was absent in the carotid body in *mdx* mice, which might explain, in part, the diminished ventilatory response to hypoxic and hypercapnic stimuli in both patients and *mdx* mice. Indeed, Burns et al. (2017) reported hypoactivity of the carotid bodies in 8‐week‐old *mdx* mice. This suggests that hypoventilation might present owing to a disruption in the afferent control of breathing that leads to reduced neural output to the effector muscles of breathing. However, subsequent studies by the same research group have demonstrated no change in basal ventilation at 4 months of age (Maxwell et al., 2023) and that normal ventilation is maintained in *mdx* mice from early through to advanced disease (O'Halloran *et al.*, 2023). Collectively, the studies suggest that there is heterogeneity in disease expression relevant to breathing in the *mdx* mouse model. Many studies have attributed ventilatory impairment in *mdx* mice to mechanical deficits in respiratory muscles owing to excessive weakness and fibrosis; however, decreased neural drive to breathe or effective neurotransmission of central respiratory drive into respiratory muscle via the neuromuscular junction might be the major contributing factor to respiratory compromise, especially in early stages of the natural history of the disease, when mechanical deficits are not evident or pronounced enough to explain ventilatory impairments (Mhandire et al., 2022; O'Halloran et al., 2023). It has not yet been established fully whether dysfunction in central chemoreceptors plays a role in the altered respiratory patterns in DMD, and this remains an important gap in our current understanding of respiratory control in muscular dystrophy. In rodents, it is essential to consider ventilatory responses to gas challenges normalized to metabolism to determine whether there is a truly inadequate ventilatory response to stimulus presentation. It is also important to distinguish altered responsiveness to a challenge, from observations of lower levels of ventilation during a challenge when baseline levels of ventilation are also reduced. Assessment of the change in ventilatory equivalent for O_2_ or CO_2_ in response to chemostimulation across the spectrum of disease progression in *mdx* mice has not yet been performed but would be useful to determine to assess ventilatory status, the timing of onset of impairments in the course of disease progression and the responses to interventional strategies.

Regardless of severe pathological changes in the *mdx* diaphragm, peak inspiratory pressure‐generating capacity is equivalent to wild‐type mice during basal breathing and during chemostimulation in 8‐week‐old *mdx* mice. In addition, peak inspiratory pressure determined during sustained tracheal occlusion is preserved. Peak performance is maintained notwithstanding that EMG activities of the principal obligatory muscles of inspiration, the diaphragm and external intercostal muscles, were significantly lower in *mdx* compared with control mice (Burns, Murphy et al., 2019). This led to the hypothesis that accessory muscles of respiration are recruited in *mdx* mice to compensate for progressive loss of force in the obligatory muscles.

It was recently established (O'Halloran et al., 2023) that peak diaphragm, external intercostal and parasternal EMG activities are much lower in *mdx* mice compared with age‐matched control animals in the early stages of disease (4 months of age), most probably owing to attenuated neuromuscular transmission (Personius & Sawyer, 2006). However, peak EMG activities of the scalene, cleidomastoid and sternohyoid muscles in *mdx* mice were equivalent to control animals, and trapezius EMG activity was greater in *mdx* mice. Peak inspiratory pressure is maintained in *mdx* mice until 12 months of age, when it declines, associated with reductions in the peak EMG activities of the accessory muscles (O'Halloran et al., 2023).

**FIGURE 3:**
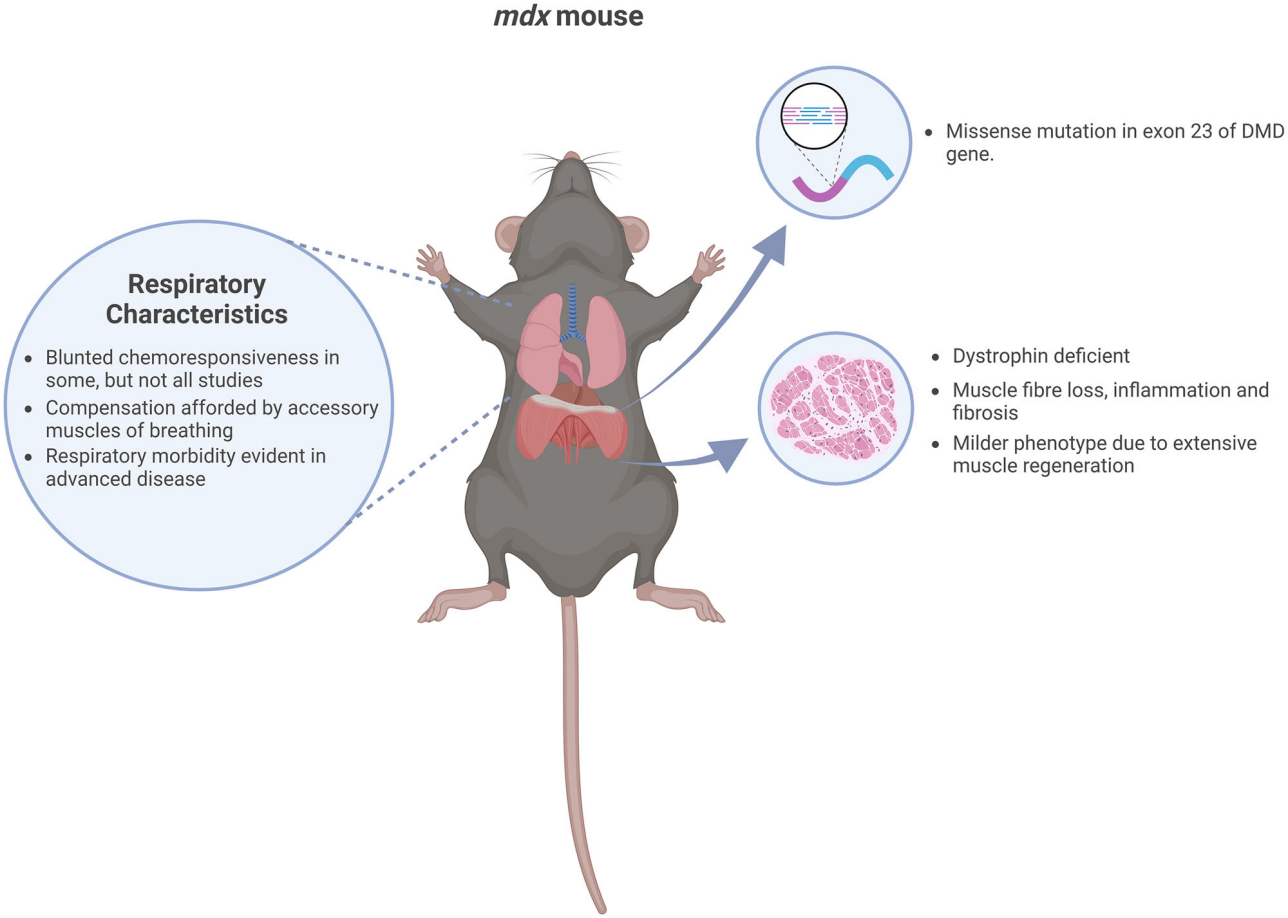
Characteristics of the *mdx* mouse model of Duchenne muscular dystrophy. Created with BioRender.com.

The study of the *mdx* mouse as a model of DMD has been transformative in our understanding of the pathophysiological features of the disease. Despite a milder phenotype in *mdx* mice compared with humans with DMD, this model has been crucial in preclinical studies of therapies for DMD, including exon skipping and gene therapy. Dystropathology of the diaphragm resembles that of human DMD, yet there is a remarkable capacity for compensation in *mdx* mice, which differs to human DMD and represents a significant limitation of the model (Figure 3). Recognition of this limitation has led to the creation of other models of DMD to mirror the human DMD phenotype better, with a view to translation from preclinical to clinical trials.

### 3.3 Dystrophin/utrophin double knockout models

It has been proposed that the milder dystrophic pathology in *mdx* mice is attributable to the upregulation of the dystrophin homologue, utrophin, which is encoded by the *utrn* gene. Utrophin functions in a similar manner to dystrophin and is present in the sarcolemma in humans during gestation, declining before birth (Clerk et al., 1993), whereas it remains in the neuromuscular junction in adult muscle. In humans with DMD, utrophin is also upregulated as a supplementary mechanism (Taylor et al., 1997). Therefore, in order to produce a more severe phenotype in the *mdx* mouse that better mirrors the human DMD phenotype, *mdx* mice were crossed with utrophin‐deficient mice (*utrn* ^−/−^) to produce double knockout *dko* mice (Deconinck et al., 1997). This mouse model expresses a more severe dystrophic phenotype, including rapid muscle degeneration, weight loss and curvature of the spine. Despite the presentation of severe pathological features in the *dko* model, their premature death before 20 weeks of age hinders their application in preclinical studies focused on the efficacy of interventions.

Following the development of the *dko* model, a more viable mouse model was created that expresses similar dystrophic features. Zhou *et al.* (2008) characterized the histopathological features of the *mdx/utrn* ^+/−^ mouse model. Inflammation and variation in the types of muscle fibres persist in limb muscles, whereas in the *mdx* model, inflammation and other pathological features diminish by 6 months of age. Increased levels of type I and IV collagen have been reported in limb muscles of *mdx/utrn* ^+/−^ mice, in comparison to *mdx* mice, which translated to poor performance in functional tests (McDonald et al., 2015). Therefore, the *mdx/utrn* ^+/−^ mouse might be a useful translatable model to test interventional therapies to alleviate the dystrophic pathology in the critical muscles of breathing.

**FIGURE 4:**
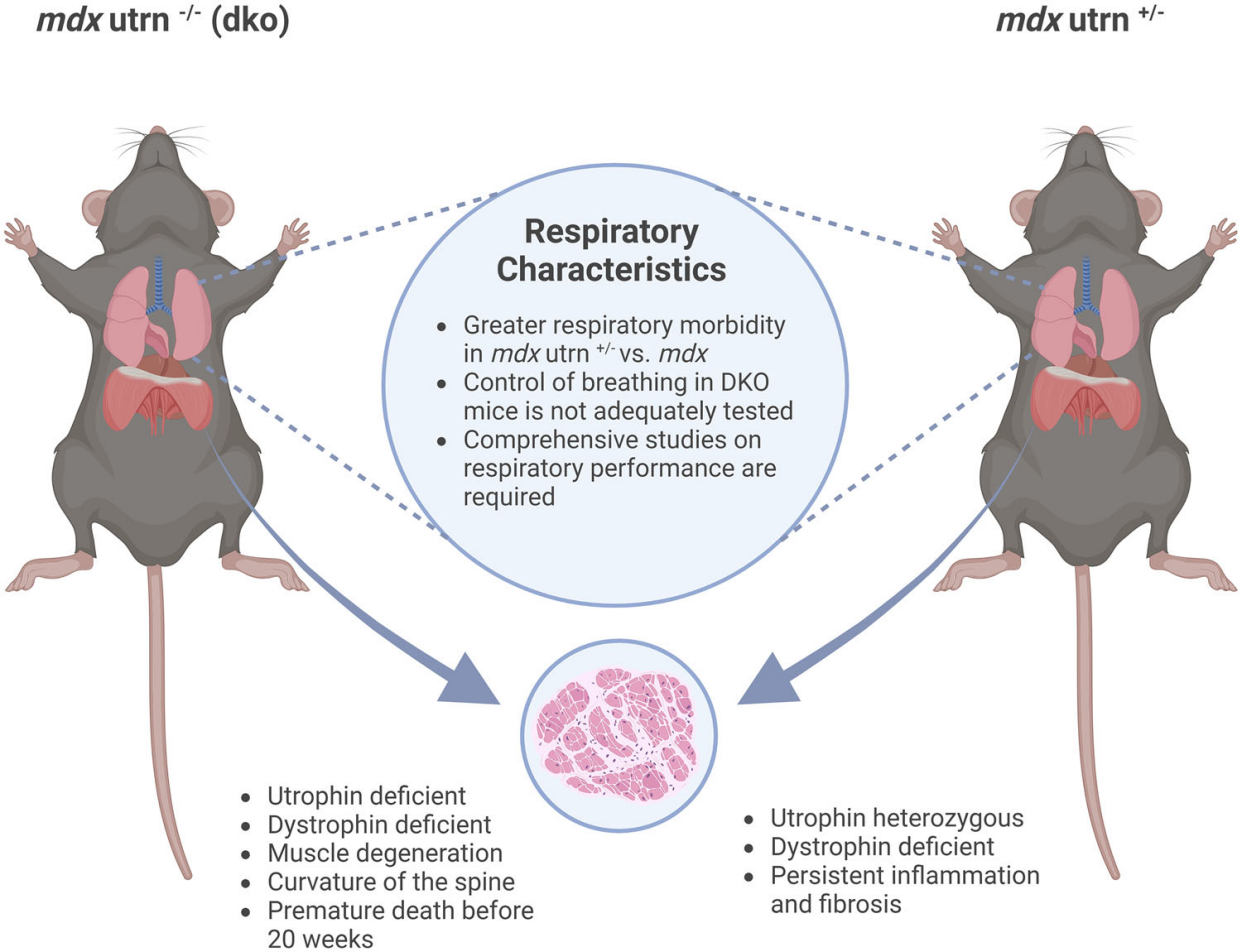
Characteristics of the *mdx utrn* ^−/−^ and *mdx utrn* ^+/−^ mouse models of Duchenne muscular dystrophy. Created with BioRender.com.

At 3 months of age, there are no significant differences in the respiratory parameters between *mdx* and *mdx/utrn* ^+/−^ mice (Huang et al., 2011). However, at 6 months, fibrosis in the diaphragm of the *mdx/utrn* ^+/−^ mice becomes more severe than that of the *mdx* mice, and the mice begin to display a more drastic pathological phenotype. Tidal volume, peak inspiratory flow and minute ventilation values are much lower in the *mdx/utrn* ^+/−^ mice than in age‐matched *mdx* mice, as expected with the increased dystropathology. Other studies have reported the more rapid and severe histopathological changes that occur in the skeletal muscle of these mice with haploinsufficiency of the utrophin gene (Gutpell et al., 2015; Zhou et al., 2008). However, there has not, to date, been a comprehensive characterization of respiratory system performance in this mouse model and, as such, it is not conclusive whether this model represents a more faithful model of human DMD (Figure 4).

A major limitation of murine models in respiratory physiology studies is the inability to measure volitional parameters, such as forced expiratory volume in 1 s and FVC (Vanoirbeek et al., 2010). These measurements are crucial for patient care and for prognostic evaluation. However, whole‐body plethysmography has been used extensively in various mouse models, providing important detail on ventilation and ventilatory responsiveness. Few studies, however, used concurrent assessment of metabolism (Burns et al., 2018, 2017; Burns, Murphy et al., 2019; Maxwell et al., 2023), which is required to draw firm conclusions relating to ventilatory insufficiency. Further assessments of peak inspiratory performance (Burns, Murphy et al., 2019; Hughes et al., 2019; O'Halloran et al., 2023) and peak respiratory electromyogram activities have been performed in mouse models of DMD, which examine reflex capacity of the respiratory neuromuscular system across the full range of behaviours from rest to peak performance.

### 3.4 Canine golden retriever muscular dystrophy (GRMD) model

Progressive muscular degeneration has been shown to arise in golden retriever dogs owing to a spontaneous mutation (Kornegay et al., 2012; Sharp et al., 1992). Only male dogs are affected, because the mutation is caused by X‐linked inheritance, like DMD in humans. These canine models display pathophysiological features that resemble the characteristics of human DMD. Fibrosis of the diaphragm in the canine model appears between birth and 6 weeks, along with clinical symptoms such as an abnormal gait and inactivity (Valentine et al., 1988). Unlike the *mdx* mouse model, muscle degeneration persists in the GRMD limb muscles, producing a much more severe pathological presentation that mimics the human phenotype. Increased respiratory rate and the involvement of the abdomen in quiet breathing have been observed in GRMD, and early death usually occurs owing to respiratory failure (Kornegay et al., 2012).

**FIGURE 5:**
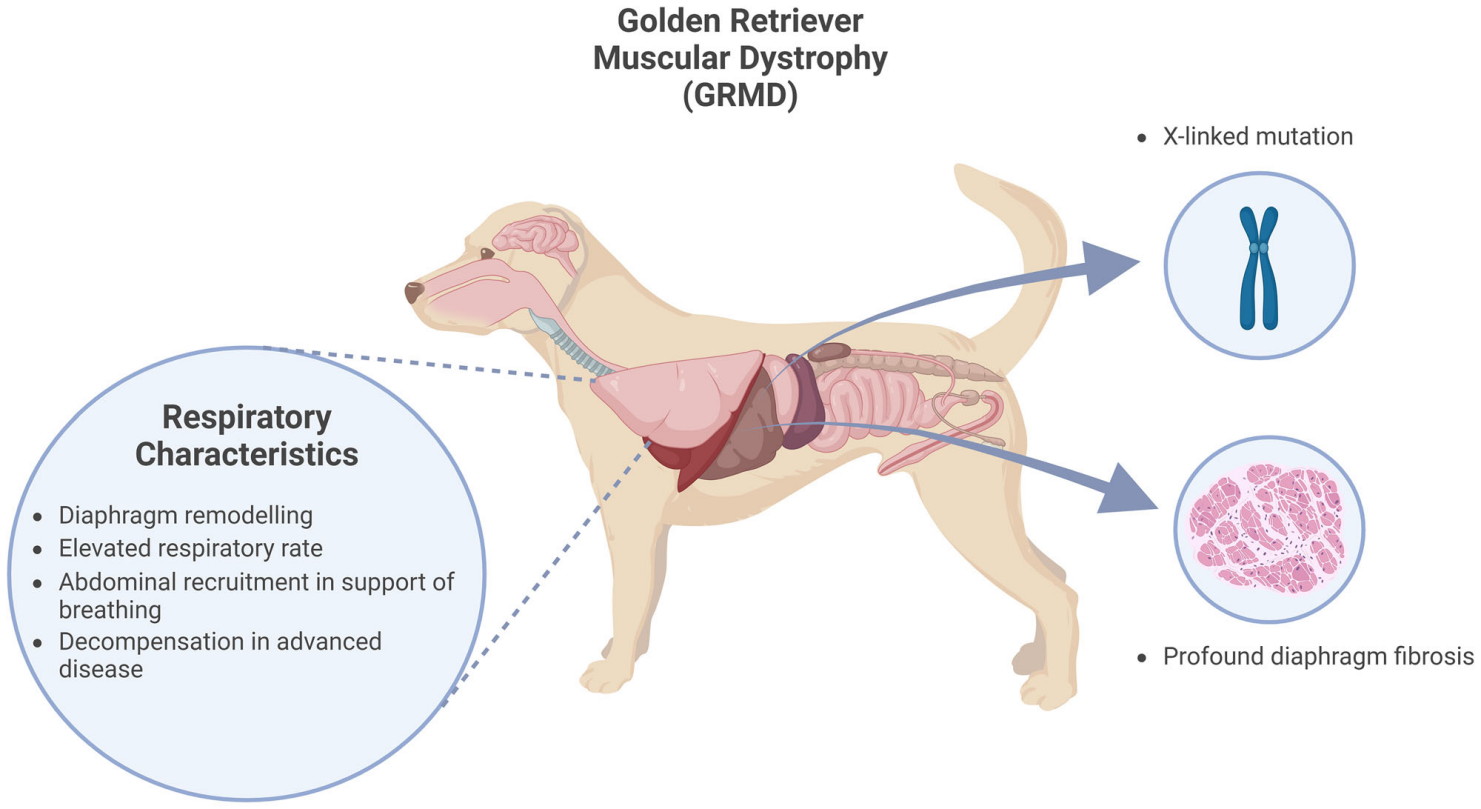
Characteristics of the golden retriever muscular dystrophy (GRMD) canine model of Duchenne muscular dystrophy. Created with BioRender.com.

Studies that aim to characterize respiration in GRMD models are limited in comparison to DMD murine models. It has been observed that GRMD dogs recruit additional expiratory muscles in the abdomen to compensate for impairments in respiration (Mead et al., 2014). In GRMD, the diaphragm is extensively fibrotic and unable to contribute to increased respiratory demand; however, there is compensatory recruitment of abdominal muscles, which raise abdominal pressure and increase expiratory flow, resulting in decreased end‐expiratory volumes and improving respiratory capacity. This is known as postexpiratory recoil and presents more commonly during periods of intense exercise or diaphragm paralysis (Grimby et al., 1976). In GRMD dogs, two peaks of abdominal movement occur for every expansion of the rib cage, occurring at early inspiration and late expiration (DeVanna et al., 2014). These observations reveal that abdominal muscles are recruited to contribute to efficient ventilation, because there were no significant differences in respiratory rate, tidal volume or minute ventilation in GRMD dogs with a median age of 47.7 months compared with control dogs. Likewise, *mdx* mice harbour the ability to compensate for diaphragm dysfunction by increased reliance on accessory muscles of respiration, including abdominal muscles (O'Halloran et al., 2023). It is reasoned that loss of compensation afforded by accessory muscles of breathing in the *mdx* mouse model leads to respiratory compromise (O'Halloran et al., 2023). Lo Mauro et al. (2010) reported that the abdominal contribution to tidal volume declines in an age‐related manner in humans with DMD (Figure 5).

Although the extent of studies characterizing respiratory system performance in GRMD models is less than in murine models, GRMD models have been used in preclinical trials for DMD treatments (Howell et al., 1997). Owing to the higher cost of canine models compared with murine models, in addition to logistical and ethical considerations, the sample sizes in these studies are typically much lower than in studies using mouse models. Owing to the accessibility of murine models, they offer greater convenience in the quest to further our understanding of the pathology of DMD; however, GRMD dogs are better suited to preclinical trials owing to the resemblance in clinical symptoms with DMD patients.

### 3.5 D2.*mdx* mouse model

Owing to the milder phenotype in *mdx* mice, different genetic strains of mice have been considered as models of DMD. Fukada et al. (2010) showed that DBA/2 mice have a significantly lower capacity for muscle regeneration owing to the striking muscle fibre loss following repeated cycles of degeneration and regeneration, in comparison to C57BL/10 mice. They hypothesized that this impairment in the self‐renewing capacity of the DBA/2 mice was attributable to a decrease in the quantity of satellite cells, which they subsequently observed in the tibialis anterior muscle following injury with cardiotoxin. They then crossed *mdx* mice with the DBA/2 mice to generate DBA/2‐*mdx* mice, which demonstrated increased fibrosis and fat accumulation in comparison to BL10‐*mdx* mice, along with fewer myofibres. In a subsequent study, Coley et al. (2016) re‐created the D2.*mdx* strain and characterized the histopathological features of these mice further at various time points.

It has been shown that DBA/2 mice carry a polymorphism in the coding region of the latent transforming growth factor‐β (TGF‐β) binding protein 4 gene (*LTBP4*) (Heydemann et al., 2009). The mutation consists of a 12‐amino‐acid deletion in *LTBP4*, which normally functions to downregulate the pro‐inflammatory cytokine TGF‐β. Inflammation greatly facilitates the pathology in skeletal muscle, leading to degeneration. Additionally, this mutation has been shown to coincide with increased fibrosis, proteolysis and SMAD signalling in DBA/2 mice (Heydemann et al., 2009). Thus, it has been hypothesized that the more severe pathological features seen in DBA/2J mice are attributable to the robust inflammatory response, because the study of muscle biopsies from humans with symptomatic DMD has shown that the TGF‐β pathway is significantly upregulated, in comparison to healthy age‐matched control subjects (Chen et al., 2005).

The histopathological markers of D2*.mdx* mice have been well characterized. Coley et al. (2016) reported that the weight of the limb muscles in D2*.mdx* mice was significantly lower than that in *mdx* mice, reflecting the atrophy in D2*.mdx* mice compared with pseudohypertrophy that occurs in *mdx* mice. The muscle atrophy in limbs of the D2*.mdx* mice is consistent with the progressive atrophy seen in humans with DMD (De Paepe, 2020). Similar results were seen in the study by Hammers et al. (2020), with the D2*.mdx* mice demonstrating significant atrophy of limb muscles between 4 and 12 months of age. In addition, fibrosis is more prominent in the D2*.mdx* diaphragm than in the *mdx* diaphragm (Coley et al., 2016; Hammers et al., 2020; Putten et al., 2019), which peaks at ∼4 months and plateaus thereafter. By 4 months of age, diaphragm force production in *mdx* mice and D2.*mdx* mice is ∼50% less than the values seen in wild‐type comparators (Hammers et al., 2020). Force production of the diaphragm remains relatively stable in D2.*mdx* mice, whereas it continues to decline in *mdx* mice with age.

Coley et al. (2016) collated data from two independent laboratories, and similar results were reported by both, showcasing that the findings are robust and reproducible. Both studies identified a reduced number of centrally nucleated fibres, which are markers of muscle regeneration, in the D2*.mdx* mice in comparison to the *mdx* mice. The impressive, long‐lasting regenerative ability of muscle in *mdx* mice is a major caveat to their use as a model of DMD. In addition, D2*.mdx* mice display persistent fibro‐adipogenic progenitors (FAPs), which arrest muscle regeneration and promote fibrosis and osteogenesis (Mázala et al., 2020). Owing to the polymorphism in *LTBP4* in D2*.mdx* mice, TGF‐β activity is enhanced, and this reduces clearance of FAPs, which is not evident in *mdx* mice (Allen & Boxhorn, 1989). The lack of regenerative capacity within the muscles of D2*.mdx* mice might be attributable to the diminished apoptosis of FAPs, which are usually present for only a short period during muscle repair, to facilitate myogenesis (Joe et al., 2010). However, their persistence within chronically damaged muscle, as seen in DMD, diminishes regeneration and leads to the secretion of extracellular matrix components (Contreras et al., 2019). Additionally, the diminished regeneration potential could be explained by increased TGF‐β signalling, which has been shown previously to inhibit myoblast differentiation into myotubes (Massagué et al., 1986).

**FIGURE 6:**
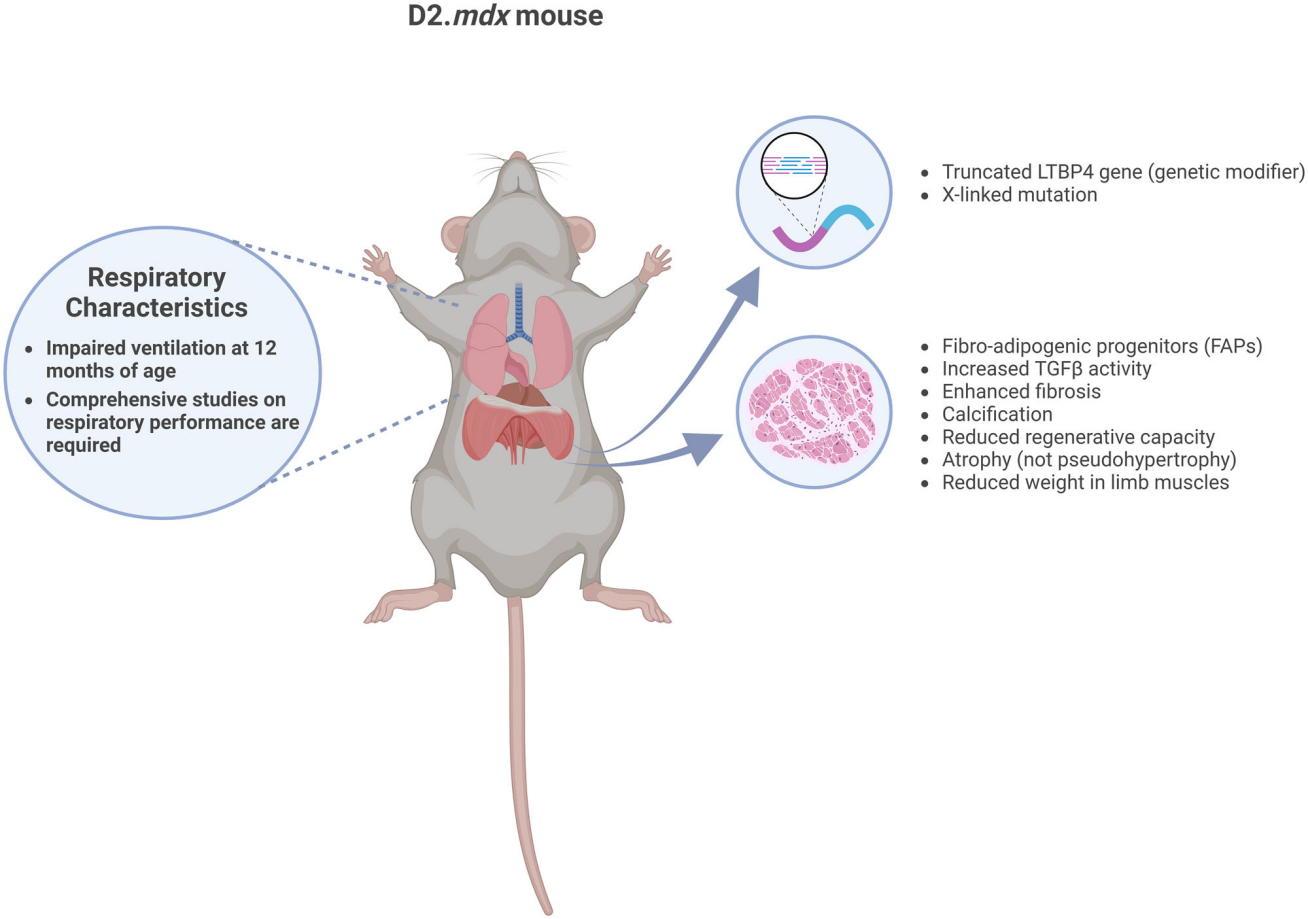
Characteristics of D2.*mdx* mouse model of Duchenne muscular dystrophy. Created with BioRender.com.

Another prominent feature of D2*.mdx* mice, which is not seen in *mdx* mice, is calcification of muscles, particularly the diaphragm, which might be facilitated by FAPs, because they can favour an osteogenic pathway (Hammers et al., 2020). Interestingly, the extent of fibrosis, calcification and muscle damage improves unexpectedly in adult D2*.mdx* mice, in comparison to younger D2*.mdx* mice (Mázala et al., 2023). Accompanying these changes is a significant decrease in the amount of FAPs, which might explain the reduction in fibrosis. However, these changes were reported in triceps muscle, and thus, might not be representative of all muscle types. The effect of enhanced fibrosis and calcified deposits in the diaphragm and accessory muscles of breathing on respiratory performance has not yet been established fully (Figure 6). One study has reported significantly impaired peak inspiratory pressure generation in 4‐week‐old D2.*mdx* mice compared with wild‐type during a tracheal occlusion challenge (Hughes et al., 2019). An early deficit in peak inspiratory pressure generation is also seen in *mdx* mice, which is compensated between 4 and 12 months before declining once again at 16 months of age (O'Halloran et al., 2023). A similar phenomenon is likely in D2.*mdx* over a shorter time frame owing to the increased dystropathology, which would make the D2.*mdx* model more convenient for study, but this needs to be determined. Characterizing respiratory performance at various time points is crucial, because changes in muscle histopathology might influence outcome measures and, as such, must be delineated clearly before assessing the efficacy of interventional therapies.

The D2*.mdx* mouse might yet prove a useful model of DMD. From recent studies, it has been demonstrated that histopathological characteristics of DMD are more pronounced than in the *mdx* mouse. Thus, understanding of the natural progression and response to therapy of this devastating disease might be enhanced by studying the biochemical and physiological features of the D2*.mdx* mouse. Trials testing the efficacy of emerging therapies often fail to focus on respiratory deficits (Mhandire et al., 2022). Of the few preclinical trials that have used D2*.mdx* mice, outcome measurements are based primarily on changes in diaphragm histology, without assessment of respiratory performance per se (Bellissimo et al., 2024; Cernisova et al., 2023). Although treatment with an adiponectin analogue, ALY688‐SR, significantly decreased the level of fibrosis in the diaphragm of D2*.mdx* mice, it remains unclear whether the intervention alters respiratory function (Bellissimo et al., 2024). In D2*.mdx* mice treated with microdystrophin, the level of collagen deposition in the diaphragm was significantly attenuated in comparison to D2*.mdx* mice that received a saline injection (Cernisova et al., 2023). Although the results from both studies are promising, given that fibrosis is a significant contributor to the pathophysiology of DMD, whether fibrosis was lessened in accessory muscles of respiration upon treatment is unclear. Ultimately, it is essential to determine the efficacy of interventional therapies to ameliorate or fully restore respiratory deficits, necessitating a comprehensive assessment of respiratory performance in preclinical models of DMD. O'Halloran et al. (2023) reasoned that as diaphragm dysfunction emerges early in DMD, and respiratory compromise appears to be related to loss of compensation by accessory muscles, targeting the accessory muscles with anti‐fibrotic or gene‐based therapies to delay or prevent respiratory failure might be more favourable, because the contribution of the diaphragm to peak pressure‐generating capacity is limited in DMD.

### 3.6 Preclinical studies assessing therapeutics for Duchenne muscular dystrophy

Systemic steroids, such as prednisone/prednisolone and deflazacort, are routinely used in the management of DMD to reduce inflammation and muscle fibre necrosis, delaying disease progression (Gloss et al., 2016). Improvements in respiratory muscle strength have been shown in boys with DMD after steroid treatment (Biggar et al., 2001; Daftary et al., 2007). Many studies have reported reduced cytokine levels and fibrosis in the diaphragm of *mdx* mice after treatment with steroids either alone or in combination with other therapies (Hartel et al., 2001; Mizunoya et al., 2011; Pereira et al., 2015). However, there is a lack of studies on the efficacy of systemic steroids to rescue breathing deficits in animal models of DMD.

Emerging therapies include both antisense oligonucleotides and gene therapy using AAVs that express a truncated form of dystrophin, such as micro‐dystrophin or mini‐dystrophin. Antisense oligonucleotides, also known as exon skipping therapeutics, exclude certain exons within mRNA located next to a mutation, to restore the reading frame, leading to dystrophin expression. There are currently four US Food and Drug Administration approved antisense oligonucleotides to treat DMD [eteplirsen (exon 51), golodirsen (exon 53), viltolarsen (exon 53) and casimersen (exon 45)], which are phosphorodiamidate morpholino oligomers (Anwar & Yokota, 2020; Clemens et al., 2020; Lim et al., 2017; Shirley, 2021). There is a paucity of information on the effects of exon skipping‐based approaches on breathing in both preclinical and clinical studies. Peptide phosphorodiamidate morpholino oligomers have been combined with additional exon skipping therapies, such as small non‐coding RNAs expressed in AAVs, to avoid vector genome loss and maintain long‐term dystrophin expression (Peccate et al., 2016). At 9 weeks of age, *dko* mice demonstrate rapid, shallow breathing, indicated by a significantly increased respiratory rate and a low tidal volume (Forand et al., 2020). A combination of phosphorodiamidate morpholino oligomer and AAV‐U7 therapy had a positive effect on respiratory function, decreasing the respiratory rate and increasing tidal volume in *dko* mice, with minute ventilation values equivalent to wild‐type mice. These effects persisted for 40 weeks and were correlated with reductions in the level of fibrosis within the diaphragm (Table 5; Forand et al., 2020).

**TABLE 5:** Effects of therapeutics on respiratory function in preclinical models of Duchenne muscular dystrophy.

Reference	Therapy	Model	Age	Outcome on respiratory function
Forand et al. (2020)	Exon skipping‐based approaches: PPMO and AAV‐U7	*Dko* (*mdx utrn* ^−/−^) mice	Three weeks old at time of cotreatment administration. Analysis at 22 and 40 weeks old	Cotreatment rescued the rapid breathing pattern in *dko* mice; decreased respiration rate and minute ventilation
Birch et al. (2023)	AAV9‐microdystrophin (μDys5) construct	GRMD dogs	Three months old at time of treatment administration. Monitored for 90 days after for analysis	Decreased peak expiratory flow‐to‐peak inspiratory flow ratio for dogs treated with the medium and high dose compared with the control group
Hayashita‐Kinoh et al. (2015)	rAAV9‐CMV‐μDys	Beagle‐based model of canine X‐linked muscular dystrophy (CXMD_J_)	Embryonic day 35 at the time of treatment administration into the amniotic fluid. One affected dog was injected again at 6 weeks old. Analysis at 70 weeks old	Reduction in respiratory rate and increase in tidal volume in systemically injected dog, values similar to normal dog. Treated dog had lower peak inspiratory flow than normal dog
Ishizaki et al. (2011)	HDAdv containing full‐length dystrophin	*Dko* (*mdx utrn* ^−/−^) mice	Seven days old at the time of treatment administration. Analysis at 8 weeks old	Significantly decreased respiratory rate and increased tidal volume in *dko* mice in comparison to untreated *dko* mice
Nelson et al. (2011)	1D11 (neutralizing antibody against transforming growth factor‐β) and losartan (an angiotensin receptor antagonist)	*Mdx* mice	Two weeks old at the time of single or cotreatment administration. Analysis at 9 months old	1D11 and losartan, alone or in combination, decreased Penh, respiratory frequency and end‐inspiratory pause
Burns et al. (2018)	Neutralizing interleukin‐6 receptor antibody (Xil‐6R) and urocortin‐2 (corticotrophin‐releasing factor receptor 2 agonist)	*Mdx* mice	Six weeks old at the beginning of treatment administration, over the course of 2 weeks	Cotreatment increased minute ventilation in *mdx* mice during normoxia. Increased ventilatory equivalent for O_2_ and CO_2_ in both wild‐type and *mdx* mice. Peak ventilatory response to hypoxia was lower in *mdx* mice and unchanged following cotreatment
Burns, Drummond et al. (2019)	*N*‐Acetylcysteine	*Mdx* mice	Six weeks old at the beginning of treatment administration, over the course of 2 weeks	No change between respiratory parameters in *mdx* and *mdx* + *N*‐acetyl cysteine group during normoxia and hypercapnic hypoxia. No change in inspiratory pressure and EMG activities during baseline and obstruction in *mdx* + *N*‐acetyl cysteine group
Selsby et al. (2016)	Quercetin	*Mdx* mice	Two months old at the time of dietary intervention. Analysis beginning at 2 months of age for a period of 12 months	During the first 6–8 months following quercetin intervention, respiratory parameters (frequency, tidal volume, minute ventilation, peak inspiratory flow and peak expiratory flow) were significantly greater in the *mdx*Q group compared with the *mdx* group that did not receive treatment. By 14 months of age, respiratory parameters were equal between the two groups
Amancio et al. (2017)	Pyridostigmine (PYR) (free and liposomal)	*Mdx* mice	Analysis at ages 6, 17 and 22 months old. Ten and 17 months old at the time of treatment administration	Pretreatment, 17‐month‐old *mdx* mice had an altered breathing pattern, showing a reduced tidal volume and increased respiratory rate, in comparison to wild‐type mice. Neither free PYR or liposomal PYR had any effect on respiratory parameters in *mdx* mice hours following administration. In 10‐month‐old *mdx* mice, respiratory parameters did not change 15 and 30 days following liposomal PYR treatment
Spaulding et al. (2020)	Quercetin‐based cocktails (quercetin, nicotinamide riboside, lisinopril and prednisolone)	*D2.mdx* mice	Four months old at the time of treatment administration. Analysis beginning at 4 months of age, every 2 months until 10 months of age	Treatment with combinations of quercetin‐based cocktails did not improve measures of respiratory function

Abbreviations: AAV, adeno‐associated virus; *dko*, double knockout; GRMD, golden retriever muscular dystrophy; HDAdv, helper‐dependent adenovirus vector; *mdx*, X‐linked muscular dystrophy mouse; PPMO, phosphorodiamidate morpholino oligomer; utrn, utrophin; μDys, microdystrophin.

Delivery of truncated yet functional forms of dystrophin using viral vectors is a promising new therapy for DMD. The full‐length dystrophin protein cannot be packaged into certain viral genomes owing to its large size. This led to the use of mini‐ and micro‐dystrophin gene therapies in an effort to alleviate dystropathology (Duan, 2018). In 3‐month‐old GRMD dogs, systemic administration of an AAV9‐microdystrophin (μDys5) construct decreased the peak expiratory flow‐to‐peak inspiratory flow ratios, measured by respiratory inductance plethysmography (Table 5; Birch et al., 2023). DeVanna et al. (2014) previously reported abnormal abdominal motion during breathing and subsequent increased peak expiratory flows in GRMD dogs, and this respiratory dysfunction was rescued by AAV‐μDys5 treatment after 90 days, in a dose‐dependent manner. Additionally, intra‐amniotic injection of rAAV9‐micro‐dystrophin in a beagle X‐linked model of DMD (CXMD_J_) demonstrated improvements in respiratory function, as shown by whole‐body plethysmography (Table 5; Hayashita‐Kinoh et al., 2015). Micro‐dystrophin rescued the rapid respiratory pattern in CXMD_J_ dogs by decreasing respiratory rate and increasing tidal volume to values comparable to the normal dog used in the study. However, peak inspiratory flow values were still significantly lower in the CXMD_J_ dogs that received micro‐dystrophin, in comparison to the control dogs. This suggests that micro‐dystrophin does not lead to a complete recovery of functional impairments in DMD but might slow the progression of the disease and produce a slightly milder phenotype.

Given that only truncated forms of dystrophin can be packaged into AAVs, various other viral genomes, such as the helper‐dependent adenovirus vector (HDAdv), have been investigated to determine their efficacy in transducing the full‐length dystrophin gene into animal models of DMD. HDAdv carrying a full‐length dystrophin complementary DNA has been shown to facilitate significant dystrophin expression, reduce the number of centrally nucleated fibres and prolong the lifespan of *dko* mice (Kawano et al., 2008). In a subsequent study, it was shown that i.p. administration of a HDAdv vector containing full‐length dystrophin improved respiratory function in *dko* mice by significantly increasing tidal volume and reducing respiratory rate (Table 5; Ishizaki et al., 2011).

Additional preclinical studies have investigated the effects of anti‐inflammatory therapeutics and antioxidants to target certain features of the pathology in DMD, which might serve as adjunctive therapies for exon skipping‐based approaches and gene therapy. Transforming growth factor‐β, a pro‐inflammatory cytokine, is elevated in DMD and has been shown to stimulate fibrosis and is therefore a major driver of the dystropathology (Kemaladewi et al., 2012). Blocking TGF‐β activity by administration of 1D11, a neutralizing antibody to TGF‐β and losartan, either alone or in combination, significantly reduced enhanced pause (Penh) in 9‐month‐old *mdx* mice, whilst also reducing fibrosis and the number of centrally nucleated fibres in the diaphragm (Table 5; Nelson et al., 2011). Cotreatment with neutralizing interleukin‐6 antibodies and urocortin‐2, a corticotrophin‐releasing factor receptor 2 agonist, in *mdx* mice demonstrated improvements in breathing (Burns et al., 2018). Minute ventilation in *mdx* mice was increased following cotreatment across a 2‐week period, resulting in significant increases in the ventilatory equivalent for both O_2_ and CO_2_. However, the peak ventilatory response to hypoxia, which is reduced in *mdx* mice, was not significantly different between *mdx* mice that received the cotreatment and *mdx* mice that received saline (Table 5). Antioxidants, such as *N*‐acetyl cysteine, have been used in preclinical studies to alleviate dystropathology (Whitehead et al., 2008). However, in *mdx* mice, *N*‐acetyl cysteine did not produce changes in respiratory parameters and had no effect on diaphragm and external intercostal baseline and maximal EMG activities, despite significant increases in diaphragm specific force and a reduction in the levels of collagen (Table 5; Burns, Drummond et al., 2019).

Quercetin, an isoflavone, has been tested in *mdx* mice owing to its ability to activate peroxisome proliferator‐activated receptor gamma coactivator 1‐alpha, and therefore, increasing the abundance of utrophin, which might compensate for dystrophin deficiency. In the initial 6–8 months of treatment with quercetin, *mdx* mice that received quercetin demonstrated a remarkable improvement in respiratory function, as indicated by significantly higher values for minute ventilation and maximal inspiratory and expiratory pressures, in comparison to *mdx* mice that did not receive treatment (Table 5; Selsby et al., 2016). However, after this time point the efficacy of quercetin began to decline, and at the 14‐month mark respiratory function was similar between *mdx* mice and *mdx* mice that received the treatment, indicating a transient but not long‐term rescue of respiratory function. Alterations in end‐plate morphology in dystrophic murine models have been reported previously (Pratt et al., 2015; Van Der Pijl et al., 2016). Given that defects at the neuromuscular junction have been hypothesized to contribute to the dystropathology of DMD, Amancio et al. (2017) administered pyridostigmine, an acetylcholinesterase inhibitor, to *mdx* mice at various time points to enhance neurotransmission. However, neither free pyridostigmine nor liposomal pyridostigmine had any effect on respiratory parameters in *mdx* mice (Table 5).

A study investigating the efficacy of quercetin, nicotinamide riboside, lisinopril and prednisolone in D2*.mdx* mice measured respiratory parameters in conscious mice using whole‐body plethysmography (Spaulding et al., 2020). The authors reported no benefit of the quercetin‐based cocktails on diaphragmatic or respiratory function. Interestingly, ventilatory insufficiency did not manifest in D2.*mdx* mice until 12 months of age, as suggested by decreased tidal volume and minute ventilation. However, metabolic measurements were not made, and therefore it remains unclear when ventilatory insufficiency presents in the model. Therefore, as a platform to develop and test therapies that target the respiratory system, complete characterization of respiratory system performance should be established in D2.*mdx* mice, mirroring the recent approach in *mdx* mice from early to advanced disease (O'Halloran et al., 2023).

## 4 CONCLUSION

Bridging the gap between preclinical research and clinical practice is crucial to treat DMD. Advances in therapies to alleviate the dystrophic pathology associated with DMD are dependent upon robust preclinical testing in animal models. Although previous murine and canine models have provided extensive knowledge surrounding pathophysiological mechanisms of DMD, a preclinical model that fully recapitulates the human DMD phenotype is yet to be determined. Data concerning the histopathological features of the novel D2.*mdx* mice are promising. In comparison to *mdx* mice, fibrosis is more striking within the diaphragm and limb muscles, and the reduction of centrally nucleated fibres in D2.*mdx* mice suggests that there is a reduced capacity for muscle regeneration, unlike *mdx* mice, which present with a milder disease phenotype (Hammers et al., 2020; Putten et al., 2019). Given that the complete delineation of respiratory performance in D2.*mdx* mice is yet to be reported, their applicability as a translational model of DMD is yet to be defined. However, it is plausible to expect that respiratory impairment is more severe in D2.*mdx* mice than in *mdx* mice, given that fibrosis is more prominent (Coley et al., 2016). Therefore, future studies should aim to characterize respiratory performance within D2.*mdx* mice comprehensively, to determine their value as a preclinical model of DMD. Such studies might provide a platform for the development of a translational pipeline testing the efficacy of established and emerging therapies for ventilatory insufficiency in human DMD.

## AUTHOR CONTRIBUTIONS

Original draft written by Rebecca Delaney, with further contribution and revisions by Ken D. O'Halloran. Both authors approved the final version of the manuscript.

## CONFLICT OF INTEREST

None declared.
